# Supplementary material for: Promoter Identification and Transcriptional Regulation of the Goose AMH Gene
Source: Animals (Basel). 2019 Oct 16;9(10):816. doi: 10.3390/ani9100816 (PMC6826907; doi:10.3390/ani9100816)
Supplement: Supplementary file 1 [file animals-09-00816-s001.pdf]

Supplemental Tables

Table S1. Primers used for cloning full-length CDS of *AMH* gene in geese.

Primer Name	sequence (5′ to 3′)	Size (bp)	*Tm* (℃)
AMH1	F: CGGCATTTGGCGTAGC<br>R: TAGCAGCGGTCCTCTTCG	257 bp	57 ℃
AMH2	F: GGACCGCTGCTACTCGCCT<br>R: CCTGGAAAGCCAGCTTAAACCA	441 bp	60 ℃
AMH3	F: ACCTGGAGGAAGTGAAGTGG<br>R: CCCGTGTTGGCTTGGAA	922 bp	60 ℃
AMH4	F: GCAGCATGAGACCGTGGA<br>R: GGGACCCTCGCAGTTGTT	712 bp	60 ℃

F: sense primers; R: antisense primers.

Table S2. Primers used for cloning the 5′-flanking sequence of *AMH* gene in geese.

Primer Name	sequence (5′ to 3′)	Size (bp)	*Tm* (℃)
AMHP1	F: GTTCCAGCGAGCGGGTGAT<br>R: CGAAACACCAGGAGGCAAGAAT	959 bp	58.4 ℃
AMHP2	F: GCCCATTATCTGTCTTTATCC<br>R: GGCAAAAGGACCCCAA	461 bp	52.0 ℃
AMHP3	F: GTCCACTTTGGCTTTGAT<br>R: GCTGGCAACACCTGAG	1599 bp	52.7 ℃
AMHP4	F: TTTCTTCCCGTGTATTGG<br>R: GAGCCAGACAGGGACAA	1457 bp	52.4 ℃

F: sense primers; R: antisense primers.

Table S3. The GenBank accession numbers of *AMH* gene sequences among various species used for sequence characterization, homology and phylogenetic analysis.

Species	Accession numbers of nucleotides and amino acids	Identities of nucleotides (%)	Identities of amino acides (%)
Anas platyrhynchos	NM_001310362.1/NP_001297291.1	95.0 %	93.9 %
Gallus gallus	NM_205030.1/NP_990361.1	85.1 %	74.4 %
Homo sapiens	NM_000479.4/NP_000470.2	47.6 %	36.8 %
Mus musculus	NM_007445.2/NP_031471.2	50.3 %	35.9 %
Bos taurus	NM_173890.1/NP_776315.1	69.4 %	36.1 %
Sus scrofa	NM_214310.2/NP_999475.2	36.8 %	37.2 %
Alligator	NM_001287280.1/NP_001274209.1	59.1 %	57.8 %
Xenopus tropicalis	XM_004911423.3/XP_004911480.1	32.6 %	31.4 %
Danio rerio	NM_001007779.1/NP_001007780.1	30.7 %	29.4 %

Table S4. Primers used for quantitative real-time PCR in geese.

Gene Name	sequence (5′ to 3′)	Size (bp)	Tm (℃)
AMH	F: TGCCCGTCCGCTATTC<br>R: CCGACAGCCGCACTCC	101 bp	60 ℃
[1]*GAPDH*	F: TTTCCCCACAGCCTTAGCA<br>R: GCCATCACAGCCACACAGA	86 bp	60 ℃
[1]*β-actin*	F: CAACGAGCGGTTCAGGTGT<br>R: TGGAGTTGAAGGTGGTCTCG	92 bp	60 ℃

F: sense primers; R: antisense primers.

[1] Housekeeping gene for data normalization.

Table S5. Primers for constructing the *AMH* promoter deletion vectors in geese.

Plasmid Name	sequence (5′ to 3′)	Size (bp)	*Tm* (℃)
pGL4.10-AMH7	F: GGGGTACCTGCATTGTCAACTCAAGGC	87 bp	50.8 ℃
pGL4.10-AMH6	F: GGGGTACCGCTGCCAACGGGAATA	331 bp	50.9 ℃
pGL4.10-AMH5	F: GGGGTACCACAGGCATTGTGAGACTTTC	637 bp	52.5 ℃
pGL4.10-AMH4	F: GGGGTACCGGAGCCATATCCCAAGC	839 bp	52.8 ℃
pGL4.10-AMH3	F: GGGGTACCTCCTCCCACTAGCAGATTAG	1452 bp	53.7 ℃
pGL4.10-AMH2	F: GGGGTACCAAGAGGAAGAGCGTTGC	1896 bp	52.6 ℃
pGL4.10-AMH1	F: GGGGTACCTCTCTATTTCTTCCCGTGTATT	2344 bp	53.2 ℃
	R: CCCTCGAGGCTCAAACGCATCTCCC		

F: sense primers; R: antisense primers.

The underline below the base indicated the protective base.

Table S6. Primers for constructing the *GATA-4* point mutation vectors in geese.

Plasmid Name	sequence (5′ to 3′)	Size (bp)	Tm (℃)
pGL4.10-AMH2-GATA4-1477	F: GTCCTCCCACTAGCACATTAGTTTTG<br>R: GTGCTAGTGGGAGGACATGCACAAT	1896 bp	55 ℃
pGL4.10-AMH2-GATA4-1399	F: CAATCATTTGGTTCACATTAAAAAAC<br>R: GTGAACCAAATGATTGTTTGAAGGA	1896 bp	55 ℃
pGL4.10-AMH2-GATA4-778	F: CATTGAGACCAAGGACATAACCTCTG<br>R: GTCCTTGGTCTCAATGTACCTGCAA	1896 bp	55 ℃

F: sense primers; R: antisense primers.

The red base represented the point mutation. The double-underline represented the GATA-box.

Supplemental Figures

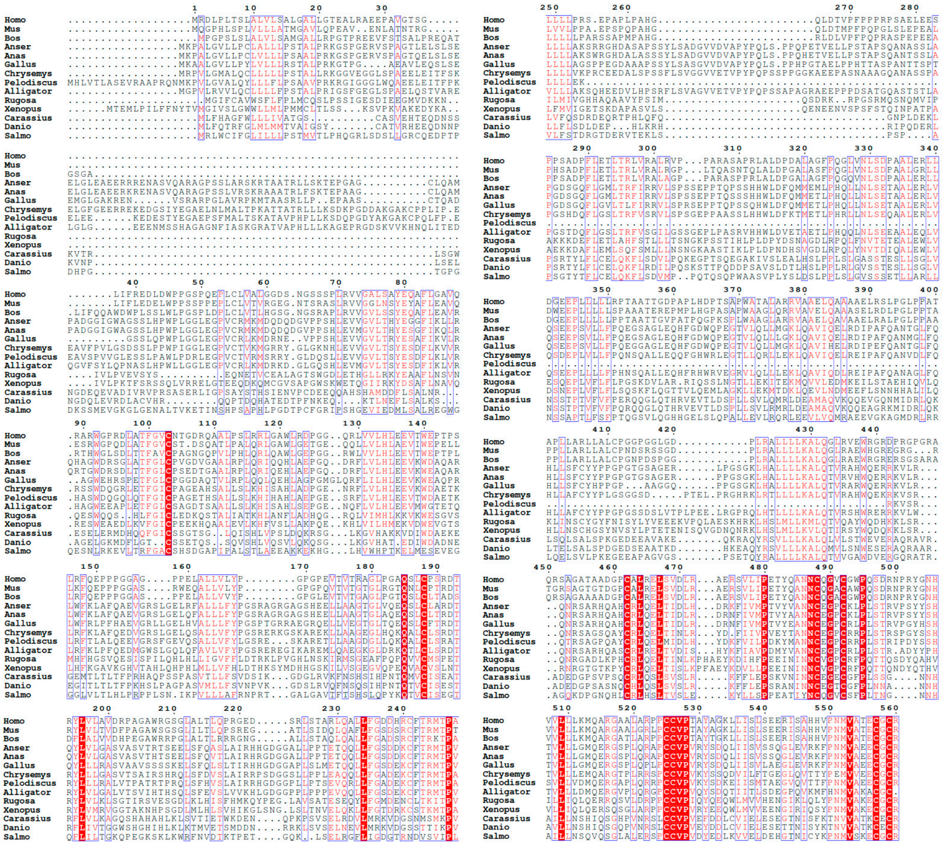

Figure S1: Homology comparison of amino acids among various species. The name of species were listed on the left side. The red background represented the consistent of amino acid. The numbers upon the sequences indicated the locations of amino acids.

-2386 CTTTCTCTATTTCTTCCCGTGTATTGGTTAAAAATGTTGCTGTGTGTTCAGTTTCTTTAGCCCTTAAATGTATAAAATGTTCCCAGTCTGGTTATATTTTGGAAGTTATCCAAAGAGGAGACAA
-2262 AGGGCTGGAAATCTCTAGGCACCTCGCCTTCAGTCAGCTACTTCTGACTGAAGTAGTTGCATCGACCTCAGCTCAGCTGTGCTAGTTTAACACATTTCCCCACCATAGACTGCTTGATGTGTA
-2139 TCTCATAAATTACCGCCAGTTAATCAGTTGCTATTCAACTGAAGTCCTTTATGAAGCAGTTCATTTGGTGATTGGGAGACTTGTCATAGACACACTTAAAGGATTTTATTACTGCATTGCCAG
-2016 TAATCCTGTTGGTGGGCAGAATGGAGGGCCAGCAGAATTTTCTACACGCAGTTTTCTGTCTTCCTGCCGAATTGCAGTTACAAGAGGAAGAGCGTTGCTCATGCTGAGTCCAGAAACCTCAT
-1894 TTCTCCTCCCGGCACTGGAGCACTGTACAGTAGCTCAGGTGTTGCCAGCTAGAAGCACGGAAATATTTCTCCTAAGGAAATCAGTTCGGACTGCTTGTCAGGAGTCCTTGAGAAGCCCTAG
-1773 ACAAGGTTCCATTGATGATTTAAAAAAAGACAATCATCCTTCCCCGAAGCGCAGCTGCCTAGCTCCTGCTTGTTAGGCTGTCTGTGCCAAATGGGGGTTCACAAACAAAGCATTGGTGCTCT
-1651 TGTCTTTGACAGTGGTAAAGGATCTCTGGGAATGTTTAGTCAGAAGAAATTACCAAAACTGCAGTTATATTAAGTCCAGGTTCGTTACTGTAATTCACTAAATGCTGTCTGTATCATTGTGA
-1529 AGCACCTGCTGTCATTAAATAGTTTTTATTGTGCATGTCCTCCCACTAGCAGATTAGTTTTGGCAAGCTTTTAGTTTCTCTGTGGTTTTGTTTATATTACATGTTTCCTTCAAACAATCATTTG
-1404 GTTCAGATTAAAAAACAGAAAATGCTCTAAGCTTTCTCCTCTCTGTCCTTTGCAGGATTCCCTTCCCCTGACGGGCGTTCCAGCGAGCGGGTGATCGCAGGGCCTCGGTGGGTGCAGGGAAG
-1282 GAGCGCCAGGCTCGGGTTGGCTCTGTGTTGGCGCCTCTTTCCACGGGCTGCCTGGGGAGGCTGAGCTTGTGTCCCGCTCCGGCCGCGTGCCCGATGGGTGGAAGGAGGCCGAGTCTTCGCC
-1161 TCTTCTGCTGGCCGGACGCAGATCTTGCCGTGTGCCCTGCAGACGTGAGAGCCAGGCAGAGTGCGTCCAGCTCCCACCTGCTTTATTGCAGCGCCGGGAGCACTTTTTCTCAATCTGAGCTG
-1039 GCCCAGTGCCTGGGATGACTTGTGTGAGCCTTCTCTCTGCCAGGTTCCTCTGCTCCCGTAGAAGAGAGGTCTGTGCTCACGAGGCCTTTGGTCTGTTGCCCTTTGTCCCTGTCTGGCTCGGGC
-916 GTGCAGTTTGGTCAGGGTCAGCCCACTCATGATGTGCTGGAGCCATATCCCAAGCTCCAAACTGCAGAAGTCCTGGGAGCCCAGAGAAAGGTCTGACTGTGCGTTGGGAACGTTTTGCAGG
-795 TACATTGAGACCAAGGAGATAACCTCTGGCAGGGCGGGGGTTTATCTGGAGTGTGTTAGGTAGGTGGTATTAGTAATTACAAGTGAGCACTGGTATCTAACAGAAGAAGTCTTTACAACAC
-674 AGGCATTGTGAGACTTTCCAAGACCTCATCTATTAAA**GCCTGAAGTATTAAAATTCATCAGGCTGCTG**CTGCACCCTGTGAGATTCTCCCTCACACATCCTGGGAGCTTTGAGATGCAGC
-554 CCAGAGCTCGGATGTGTTAAGAGCCTCTGTAAGGCGGCCTCTACACCACGGTGCACAGGGTGCCAGCCGAGGGGGAGGCTTTCCAGCAAAGAGGGAGCTTTCTATACATTTACTTAGTTTC
-433 TGCCCATTATCTGTCTTTATCCCCCTCCTCCAAGTCTAGCCAATTCTTGCCTCCTGGTGTTTCGCTGCCAACGGGAATAGTATGTGCCAGTCCCCATGCAGA**TTCAAGTCTAGACTTCCACC**
-311 **CTCCTTTAAAAAACATCTGAAGTCAAGGACGTGCATCCCTACAAGTAGGAGAAACAGCTTTAATTTTTTTTTTTTTAATTTCAATACTGGATGCAAACCCAAAAATAGAACAGAAAA**
-195 **GTTTCTTTGAAACATCCCTCCCTCCTTTCCAGCATGACAGTGTTTGAGTTTCTCAAGGGAGGTGTGTTGTGCATTGTCAACTCAAGGCCACCGGTTTTTAAAAGGGAGA**TGCCGTC
-67 CTCCCCTTCCGCGCAAATGTTTTGGGAGATGCGTTTGAGCAGCTCAAACAACGGCATTTGGCGTAGCTGAAGCGGGAAC**ATG**

Figure S2: The promoter region of goose *AMH* gene. The promoter sequence was numbered on the left side. The red letters represented the five core promoter transcription regions. The red boxes represent the TATA-box. The red underline represents the consensus sequence of GATA-4 binding site/GATA-box. The green boxes represent the GATA-box. The underline and blot font represents the identified key region which essential regulatory element locates in.

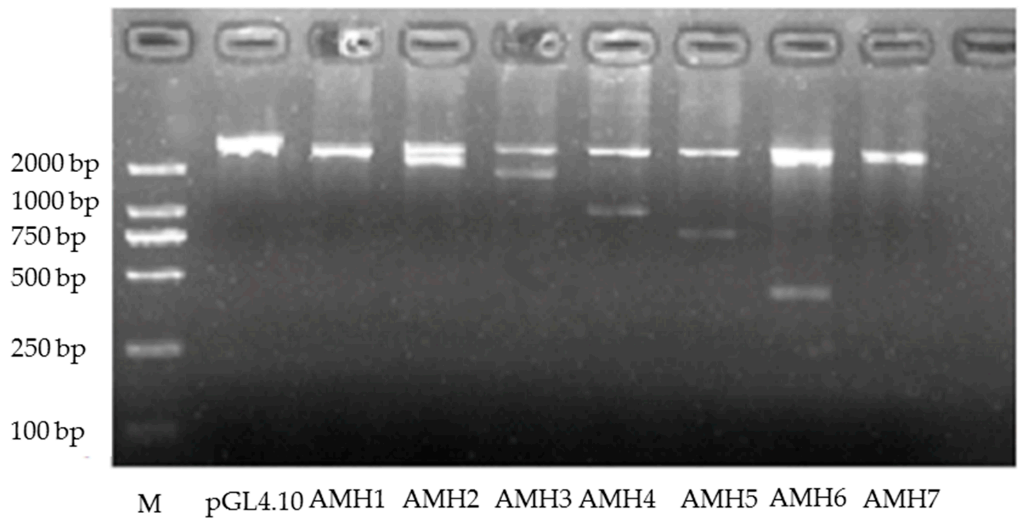

Figure S3: The construction of deletion reporter vectors. The numbers located on the left side represented the size of marker. 'M' represented the marker. pGL4.10 represented the control. AMH1 ~ AMH7 represented the constructions of deletion reporter vectors.

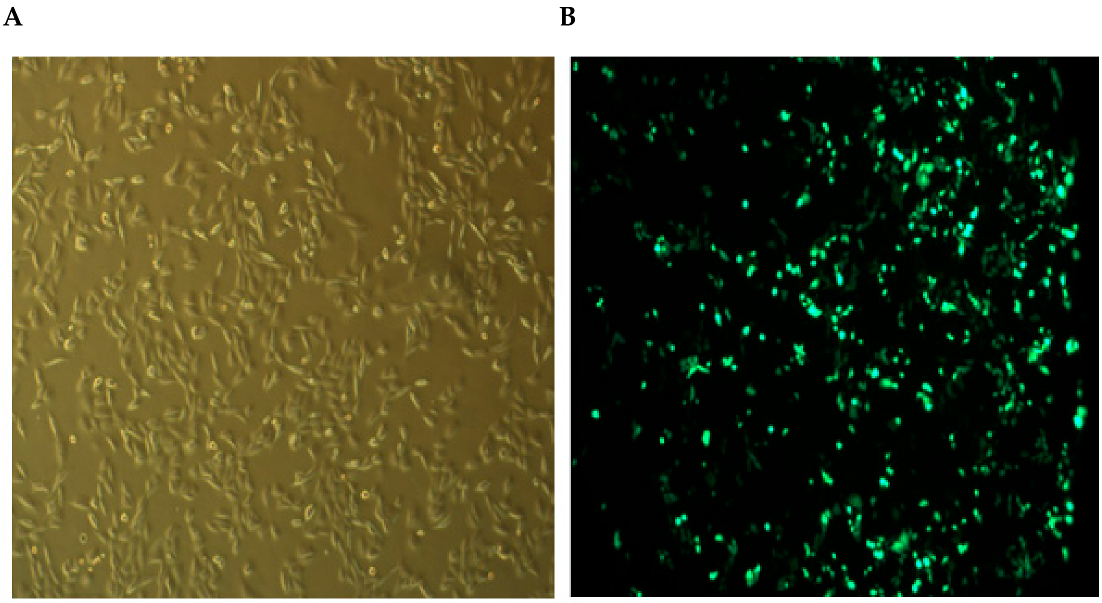

Figure S4: The transfection efficiency of GATA-4 overexpression in CHO cells. (A) The morphology of CHO cells before transfection of GATA-4. (B) The detection of green fluorescence after transfection of GATA-4.
